# Determinants of uptake of malaria preventive interventions among pregnant women in eastern Uganda

**DOI:** 10.1186/s12936-020-03558-1

**Published:** 2021-01-03

**Authors:** Solomon Tsebeni Wafula, Hilbert Mendoza, Aisha Nalugya, David Musoke, Peter Waiswa

**Affiliations:** 1 Department of Disease Control and Environmental Health, Makerere University School of Public Health, Kampala, Uganda; 2 Department of Epidemiology and Social Medicine, Faculty of Medicine and Health Sciences, University of Antwerp, Antwerp, Belgium; 3 Department of Health Policy, Planning and Management, Makerere University School of Public Health, Kampala, Uganda

**Keywords:** ITN use, IPTp3 uptake, Malaria preventive interventions, Pregnant women, Eastern Uganda

## Abstract

**Background:**

Consistent use of insecticide-treated nets (ITNs) and intermittent preventive treatment in pregnancy (IPTp) have been recommended as cost-effective interventions for malaria prevention during pregnancy in endemic areas. However, the coverage and utilization of these interventions during pregnancy in sub-Saharan Africa is still suboptimal. This study aimed to determine the uptake of IPTp and ITNs and associated factors among women during their recent pregnancy in Eastern Uganda.

**Methods:**

This was a cross-sectional study conducted among 2062 women who had delivered within the last 12 months prior to the start of the study in three districts of Eastern Uganda. The primary outcomes were consistent ITN use and optimal uptake (at least 3 doses) of IPTp. A modified Poisson regression was used to examine the association between consistent ITN use and the uptake of optimal doses of IPTp with independent variables. Data were analysed using Stata 14 software.

**Results:**

The level of uptake of IPTp3 (at least three doses) was 14.7%, while IPTp2 (at least two doses) was 60.0%. The majority (86.4%) of mothers reported regularly sleeping under mosquito nets for the full duration of pregnancy. Uptake of IPTp3 was associated with engaging in farming (adjusted PR = 1.71, 95% CI [1.28–2.28]) or business (adjusted PR = 1.60, 95% CI [1.05–2.44]), and attending at least 4 antenatal care (ANC) visits (adjusted PR = 1.72, 95% CI [1.34–2.22]). On the other hand, consistent ITN use was associated with belonging to the fourth wealth quintile (adjusted PR = 1.08, 95% CI [1.02–1.14]) or fifth wealth quintile (adjusted PR = 1.08, 95% CI [1.02–1.15]), and attending at least 4 ANC visits (adjusted PR = 1.07, 95% CI [1.03–1.11]).

**Conclusion:**

Uptake of IPTp3 and consistent ITN use during pregnancy were lower and higher than the current Ugandan national targets, respectively. Study findings highlight the need for more efforts to enhance utilization of ANC services, which is likely to increase the uptake of these two key malaria preventive measures during pregnancy.

## Background

Pregnant women are a high-risk group for malaria and may have adverse consequences if infected. Malaria during pregnancy increases the risk of maternal and fetal anaemia, low birth weight, stillbirth, spontaneous abortion, and neonatal death [1]. The World Health Organization (WHO) estimates that 11 million pregnancies in moderate and high malaria transmission sub-Saharan African countries such as Uganda were exposed to malaria infection in 2018 [2]. Consequently, about 872,000 children were born with low birth weight and more than 40% of maternal anaemia cases due to malaria occurred in these countries [2]. In studies in Uganda, malaria prevalence among pregnant women ranges from 13.1 to 50.0% [3, 4], but can be as high as 80% in highly endemic regions [5].

The WHO recommends a three-pronged package consisting of intermittent preventive treatment in pregnancy (IPTp) with sulfadoxine-pyrimethamine (SP), use of insecticide-treated bed nets (ITNs), and effective case management of clinical malaria and anaemia in areas with moderate to high malaria transmission rates [2]. In highly malaria-endemic countries such as Uganda, intermittent preventive treatment in pregnancy with SP (IPTp-SP) is recommended to prevent the adverse consequences of malaria on maternal and fetal outcomes. In 2012, the WHO updated its recommendations on IPT-SP and now requires that at least three doses of SP be given to all pregnant women at each scheduled antenatal care (ANC) visit starting as early as possible in the second trimester and given at one-month intervals [6]. In Uganda, the target is to have 93% of pregnant women receive at least two doses of SP during antenatal care (ANC) visits, and 80% to receive at least 3 doses (optimal doses). The use of ITNs is also recommended for all pregnant women in malaria-endemic countries, and in Uganda, the Malaria Reduction Strategic Plan 2014–2020 set targets for 100% of the population including pregnant women to be distributed with ITNs, and up to 85% to be sleeping under ITNs the previous night by the end of 2020 [7].

Despite these set national targets, the uptake of at least three doses (optimal dose) of IPTp-SP in Uganda is low at 18% [8]. Regarding ITN use, 90% of households owned at least one ITN but 75% of pregnant women aged 15–49 years reported having slept under an ITN the previous night, according to the latest malaria indicator survey [9]. Utilization of these interventions may be more challenging in rural districts of the country due to poor access to health facilities and lack of information on these strategies [10]. This justifies the need to examine factors that could contribute to this underachievement. A review of literature of factors associated with utilization of these preventive measures in sub-Saharan Africa highlighted both health system related factors (anti-malarial drug stockouts, limited safe water at ANC clinics, unavailability of skilled attendants, poor health personnel attitudes and unavailability of ITNs) and individual factors (woman’s knowledge, pregnancy and woman’s economic and social position and religious practices) [11, 12]. However, there exists geographical variation (between country and in country differences) of these predictors thus justifying the need for this study. There is limited information on individual factors associated with the utilization of IPTp and ITNs in rural Uganda. The Eastern region of Uganda has one of the highest malaria prevalence rates in the country [9]. Understanding these factors can increase the uptake of IPTp and ITNs during pregnancy, and consequently reduce the malaria burden in Uganda. This study aimed to assess the level of uptake of IPTp and ITNs and associated factors among women during their recent pregnancy in Eastern Uganda.

## Methods

### Study design and settings

This was a cross-sectional study conducted in the districts of Iganga, Luuka and Buyende in eastern Uganda. This region is predominantly rural, cover an area of 3549.8 km^2^, and have an estimated population of 1,065,284 inhabitants living in 208,030 households [13]. These districts are served by at least 75 government-run health facilities and several private not for profit (PNFP) health centres [14]. Malaria, which is mostly attributable to *Plasmodium falciparum*, is endemic in this area. The main economic activity in these districts is subsistence farming, but other occupations include small-scale businesses, such as fishing, grain milling, market vending, motorcycle transport and formal employment. The Basoga, a Bantu-speaking group, are the predominant ethnic group, which make up to 9% of Uganda’s population [14].

### Study domain, eligibility and sampling

The study units were households, and the study domain included women who had delivered in the last 12 months prior to the start of the study and were resident in the area. Mothers were included in the study whether the child was delivered preterm or full-term, and irrespective of the birth outcome (whether the baby was alive or dead). Those who had not lived in the community for at least 1 year were excluded from the study.

Data were collected from 2062 mothers in three health sub-districts (HSDs): Buyende, Luuka, and Iganga. Sixteen (16) sub-counties (6 in Buyende, 6 in Luuka, and 4 in Iganga) were proportionately selected from the HSDs. The sub-counties in each HSD were randomly selected and within each sub-county, one parish was randomly selected. Two villages were randomly selected from each parish, and a list of households with mothers who met the criteria were listed. Participants were sampled at the village level using simple random sampling from the village listing made with the aid of local council 1 (village) leader. From each selected village, at least 50 households were visited by the enumerators from which one eligible respondent was selected per household.

### Data collection and study variables

This study utilized secondary data from a broader study entitled “Innovations for increasing access to integrated safe delivery, PMTCT and newborn care in Rural Uganda”. An interviewer-administered structured questionnaire developed based on the literature on the uptake of IPTp-SP and ITNs among pregnant women was used to collect quantitative data. The original English questionnaire was translated to *Lusoga*, the local language spoken by the study participants. Data were collected on socio-demographic characteristics, uptake of IPTp-SP, ITNs, and frequency of ANC visits. Research assistants were trained on appropriate methods of data collection, and the tool appropriately piloted. The primary outcome variables of the study were consistent ITN use and optimal uptake of IPTp-SP which were self-reported. Consistent ITN use was defined as sleeping under an ITN every night for the full duration of the last pregnancy, while optimal uptake of IPTp-SP was defined as 3 or more doses received during pregnancy. The covariates (independent variables) included the timing of first ANC, number of ANC visits, sociodemographic characteristics (such as maternal age, marital status, level of education of women, occupation, household size, parity, and wealth (measured using a wealth asset index). The wealth quintiles were generated using principal component analysis based on the information collected on assets owned and household structure. The covariates used for this study were selected from critical review of related published literature [11, 12].

### Statistical analysis

Data were analysed using Stata Version 14.0 (StataCorp, Texas, US). Descriptive statistics such as frequencies and percentages were used to present categorical data, while means and standard deviations were used where data were continuous. The associations between the outcome variables (consistent ITN use and uptake of 3 or more IPTp-SP doses) and explanatory variables were explored using modified Poisson regression. Initially, unadjusted prevalence ratios (PRs) were obtained for the association between each outcome and each predictor variable. Prevalence ratios were preferred over odds ratios since odds ratios would overestimate the effect size when outcomes are common (prevalence > 10%) [15, 16], as was the case in the current study. All epidemiologically meaningful independent variables were considered for a fully saturated model. Interactions between predictor variables and the primary outcomes were as well examined. A stepwise backward elimination method was then applied, removing variables with the largest non-significant *p* values, systematically until only significant variables and those that improved the fit of the model were retained. The prevalence ratios (PR) and 95% confidence intervals are presented. A *p*-value of less than 0.05 was considered statistically significant.

## Results

### Background characteristics of participants

A total of 2062 women who had been pregnant in the past 12 months within 1 year preceding the survey participated. The mothers’ ages ranged from 14 to 49 years, with a mean age of 25.8 years (SD ± 6.6). About three-quarters of the participants, 1280 (62.1%) had primary school as their highest level of education, and the majority 1894 (91.9%) were married or cohabitating. More than half of all women 1101 (53.4%) made their first antenatal care (ANC) visit after 12 weeks of gestation, while 1309 (63.5%) had at least 4 ANC visits throughout the gestation period (Table 1).

**Table 1:** Socio-demographic characteristics of the study participants

Characteristic	Number of participants (N = 2062)	Percentage (%)
Age in years		
< 20	353	17.1
20–24	623	30.2
25–29	452	21.9
30–34	296	14.3
≥ 35	226	11.0
Not stated	112	5.4
Highest education level		
None	248	12.0
Primary	1280	62.1
Post primary	523	25.4
Not stated	11	0.5
Parity		
Primiparous	362	17.6
2–4	916	44.4
≥ 5	776	37.6
Not stated	8	0.4
Marital status		
Married	1894	91.9
Not married	168	8.1
Occupation		
Farmer	1218	59.1
Housewife	554	26.9
Business	181	8.8
Other^a^	109	5.3
Timing of first ANC visit (weeks)		
≤ 12	947	45.9
> 12	1101	53.4
Not stated	14	0.7
Number of ANC visits		
1–3	710	34.4
≥ 4	1309	63.5
Not stated	43	2.1

*ANC* antenatal care

^a^Other occupations included salaried work, casual labourers

### Utilization of malaria preventive strategies during pregnancy

The majority of participants 1904 (92.3%) reported using ITNs at least once, whereas 86.4% (1772/2052) reported using ITNs regularly for the full duration of their last pregnancy. Consistent ITN use was significantly higher in Iganga district (89.8%), followed by Buyende (86.7%), then Luuka district (83.3%) (*p*-value = 0.007). Regarding IPTp uptake, 289 (14.7%) of the participants had used at least 3 doses of IPTp-SP, 1178 (60.0%) received at least two doses, 1603 (81.6%) at least one dose, and 361 (18.4%) did not receive any dose of IPTp-SP during their last pregnancy.

### Predictors of use of ITNs during pregnancy

The proportion of women who regularly used ITNs during their last pregnancy was 7% lower in Luuka compared to Iganga district (adjusted PR = 0.93, 95% CI [0.88–0.97]). Women belonging to the fourth (adjusted PR = 1.08, 95% CI [1.02–1.14]) and fifth (highest) (adjusted PR = 1.08, 95% CI [1.02- 1.15]) wealth quintiles were each 1.08 more likely to report using ITNs regularly during pregnancy compared to those in the lowest quintile. Women who had at least 4 antenatal care visits had a 7% higher likelihood of regularly sleeping under ITNs during pregnancy compared to those who had fewer than 4 visits (adjusted PR = 1.07, 95% CI [1.03–1.11]) (Table 2).

**Table 2:** Crude and adjusted analysis of predictors of consistent ITN use during pregnancy

Characteristic	Uptake of ITNs n (%)		Crude PR [95% CI]	*p*-value	Adjusted PR [95% CI]	*P*-value
	Consistent	Not consistent				
District						
Iganga	360 (90.2)	39 (9.8)	1		1	
Luuka	686 (83.8)	133 (16.3)	0.93 [0.89–0.97]	0.001	0.93 [0.88–0.97]	*0.001*
Buyende	723 (87.0)	108 (13.0)	0.96 [0.92–1.00]	0.087	0.98 [0.94–1.02]	0.386
Age in years						
< 20	295 (84.0)	56 (16.0)	1			
20–24	516 (83.5)	102 (16.5)	0.99 [0.94–1.05]	0.823		
25–29	406 (89.8)	46 (10.2)	1.07 [1.01–1.13]	0.018		
30–34	263 (89.5)	31 (10.5)	1.06 [1.00–1.13]	0.042		
≥ 35	198 (88.0)	27 (12.0)	1.05 [0.98–1.12]	0.175		
Marital status						
Married	1635(86.7)	250 (13.3)	1			
Not married	137 (82.0)	30 (18.0)	0.95 [0.88–1.02]	0.135		
Education level						
None	208 (84.2)	39 (15.8)	1			
Primary	1099 (86.2)	176 (13.8)	1.02 [0.97–1.09]	0.433		
Post primary	455 (87.7)	64 (12.3)	1.04 [0.98–1.11]	0.210		
Husband education level						
None	215 (83.7)	42 (16.3)	1			
Primary	827 (87.1)	122 (12.9)	1.04 [0.98–1.11]	0.177		
Post primary	617 (86.9)	93 (13.1)	1.04 [0.98–1.10]	0.223		
Parity						
Primiparous	298 (83.0)	61 (17.0)	1			
2–4	782 (85.8)	129 (14.2)	1.03 [0.98–1.09]	0.221		
≥ 5	685 (88.5)	89 (11.5)	1.06 [1.01–1.12]	0.018		
Occupation						
Housewife	483 (87.5)	69 (12.5)	1			
Peasant farmer	1040 (86.0)	170(14.0)	0.98 [0.94–1.02]	0.368		
Business	156 (86.2)	25 (13.8)	0.99 [0.92–1.05]	0.655		
Other occupation	93 (85.3)	16 (14.7)	0.98 [0.90–1.06]	0.556		
Household size						
1–4	606 (83.8)	117 (16.2)	0.96 [0.91–1.01]	0.112		
5–8	863 (87.8)	120 (12.2)	1.00 [0.96–1.05]	0.847		
> 8	298 (87.4)	43 (12.6)	1			
Wealth index						
1 (lowest)	338 (83.5)	67 (16.5)	1		1	
2	363 (85.4)	62 (14.6)	1.02 [0.97–1.09]	0.438	1.03 [0.97–1.09]	0.325
3	368 (85.6)	62 (14.4)	1.03 [0.97–1.09]	0.397	1.03 [0.97–1.10]	0.271
4	334 (88.6)	43 (11.4)	1.06 [1.00–1.12]	0.038	1.08 [1.02–1.14]	*0.009*
5 (highest)	342 (89.5)	40 (10.5)	1.07 [1.02–1.13]	0.013	1.08 [1.02–1.15]	*0.010*
Timing of first ANC visit (weeks)						
≤ 12	834 (88.4)	109 (11.6)	1			
> 12	928 (84.8)	167 (15.3)	0.96 [0.93–0.99]	0.014		
Number of ANC visits						
1–3	586 (83.0)	120 (17.0)	1		1	
> 4	1154 (88.6)	149(11.4)	1.07 [1.03–1.11]	0.001	1.07 [1.03–1.11]	*0.001*

Italic values indicate the significance of p-value (p-value < 0.05)

*ANC* antenatal care, *CI* confidence interval, *PR* prevalence ratio

### Predictors of optimal uptake of IPTp-SP during pregnancy

Multivariable regression showed that women who were farmers (Adjusted PR = 1.71, 95% CI [1.28–2.28]) and those engaged in business (Adjusted PR = 1.60, 95% CI [1.05–2.44]) were respectively 1.7 and 1.6 times more likely to receive at least 3 doses of IPTp-SP compared to housewives. Women who had attended at least 4 ANC visits had a 70% higher chance of receiving at least 3 doses of IPTp-SP compared to those who only attended ANC 3 times or less (Adjusted PR = 1.72, 95% CI [1.34–2.22]) (Table 3).

**Table 3:** Predictors of uptake of 3 or more doses of IPTp-SP during pregnancy

Characteristic	Uptake of IPTp-SP		Crude PR [95% CI]	p-value	Adjusted PR [95% CI]	P-value
	≥ 3 doses (%)	< 3 doses (%)				
District						
Iganga	51 (13.4)	330 (86.6)	1			
Luuka	147 (18.6)	642 (81.4)	1.39 [1.04–1.87]	0.028		
Buyende	91 (11.5)	701 (88.5)	0.86 [0.62–1.18]	0.350		
Age in years						
< 20	48 (14.0)	294 (86.0)	1			
20–24	93 (15.6)	503 (84.4)	1.11 [0.81–1.53]	0.519		
25–29	69 (15.9)	365 (84.1)	1.13 [0.81–1.59]	0.472		
30–34	26 (9.4)	253 (90.6)	0.67 [0.42–1.05]	0.077		
≥ 35	27 (12.7)	185 (87.3)	0.91 [0.58–1.41]	0.665		
Marital status						
Married	259 (14.4)	1546 (85.6)	1			
Not married	30 (18.9)	129 (81.1)	1.31 [0.93–1.85]	0.1161		
Education level						
None	27 (11.6)	206 (88.4)	1			
Primary	175 (14.3)	1049 (85.7)	1.23 [0.84–1.80]	0.279		
Post primary	87 (17.5)	410 (82.5)	1.51 [1.01–2.26]	0.045		
Husband education level						
None	28 (11.7)	212 (88.3)	1			
Primary	122 (13.5)	782 (86.5)	1.16 [0.79–1.70]	0.459		
Post primary	117 (16.9)	575 (83.1)	1.45 [0.99–2.13]	0.059		
Parity						
Primiparous	52 (15.1)	293 (84.9)	1			
2–4	147 (16.7)	733 (83.3)	1.11 [0.83–1.48]	0.488		
≥ 5	90 (12.3)	642 (87.7)	0.82 [0.59–1.12]	0.207		
Occupation						
Housewife	52 (9.8)	481 (90.2)	1		1	
Peasant farmer	193 (16.7)	961 (83.3)	1.71 [1.28–2.29]	< 0.001	1.71 [1.28–2.28]	*< 0.001*
Business	28 (16.3)	144 (83.7)	1.67 [1.09–2.56]	0.019	1.60 [1.05–2.44]	*0.031*
Other occupation	16 (15.2)	89 (84.8)	1.56 [0.93–2.62]	0.093	1.47 [0.86–2.52]	0.158
Household size						
1–4	108 (15.5)	589 (84.5)	1.25 [0.89–1.75]	0.194		
5–8	141 (15.0)	799 (85.0)	1.21 [0.87–1.68)]	0.252		
> 8	40 (12.4)	283 (87.6)				
Wealth index						
1 (lowest)	49 (12.7)	336 (87.3)	1			
2	56 (13.7)	354 (86.3)	1.07 [0.75–1.53]	0.699		
3	52 (12.4)	368 (87.6)	0.97 [0.68–1.40]	0.882		
4	52 (14.5)	306 (85.5)	1.14 [0.79–1.64]	0.475		
5 (highest)	71 (19.7)	289 (80.3)	1.55 [1.10–2.17]	0.010		
Timing of first ANC visit (weeks)						
≤ 12	152 (16.8)	753 (83.2)	1			
> 12	137 (13.0)	921 (87.1)	0.77 [0.62–0.95]	0.017		
Number of ANC visits						
1–3	69 (10.0)	620 (90.0)	1		1	
4+	216 (17.2)	1040 (82.8)	1.72 [1.33–2.22]	< 0.001	1.72 [1.34–2.22]	*< 0.001*

Italic values indicate the significance of p-value (p-value < 0.05)

*ANC* antenatal care, *CI* confidence interval, *PR* prevalence ratio

## Discussion

This study investigated the uptake of IPTp and ITNs, and associated factors among women during their recent pregnancy in Eastern Uganda. Findings show that the level of uptake of IPTp3 (14.7%) was lower than that of consistent ITN use (86.4%). The study also revealed that consistent ITN use was associated with wealth index and ANC attendance of at least 4 times while uptake of IPTp3 was influenced by mother’s occupation and ANC attendance of at least 4 times.

While the study indicated high levels of ANC attendance, ≥ 4 visits (63.5%), the uptake of optimal doses of IPTp (14.7%) was lower than the national target of 80% [7] and current national coverage of 18.5% [8]. These findings concur with those of a study conducted in Tanzania, where only 11% of the women had received optimal doses of IPTp during pregnancy [17]. However, higher coverage was reported in a study conducted in Ghana, where 71% of the pregnant women received the optimal doses of IPTp [18]. The low uptake indicated in this study could be due to missed opportunities to administer IPTp due to regular stock-out of SP, which has been reported in the literature as one of the major barriers to IPTp uptake at health facilities in Uganda [11]. In addition, sub-optimal ANC attendance by pregnant women could contribute to the low utilization of IPTp. For instance, pregnant women attending ANC only during the first trimester when IPTp is not supposed to be administered affects uptake of the intervention. Therefore, such health systems barriers to ANC need to be addressed which in turn are likely to increase utilization of IPTp.

A significant proportion of participants (92.3%) reported using ITNs at least once during their last pregnancy, and 86.4% reported using ITNs regularly during the entire pregnancy. The high uptake of ITNs in this study could be attributed to existing strategies in Uganda such as ITN mass distribution campaigns among high-risk populations including pregnant women. Existing data indicate that utilization and ownership of ITNs has substantially improved owing to such campaigns in many countries in sub-Saharan Africa [19]. Similar findings were shown in a study conducted in the Democratic Republic of Congo, where 78.2% reported sleeping under ITNs regularly [20]. Lower rates of usage were however reported in a study conducted in Gulu district in Northern Uganda where only 35% of the pregnant women used ITNs [21]. This low utilization of ITNs in Northern Uganda could be because the study was conducted in an internally displaced peoples (IDP) camp, where the exchange and selling of ITNs to meet other immediate basic needs such as food is a common practice [21, 22]. Increased national campaigns on the use of ITNs by the Ministry of Health and other stakeholders is likely to further increase their utilization including among pregnant women in Uganda.

In this study, women who had at least 4 ANC visits had a 7% higher likelihood of consistently sleeping under ITNs during pregnancy compared to those who had less than 4 visits. These findings are similar to those of a study conducted in Ethiopia where the number of ANC visits was found to be significantly associated with the utilization of ITNs [23]. This finding could be because pregnant women who frequently attend ANC are likely to be exposed to vital health related information on ITN usage, which leads to regular use of ITNs. Attending ANC also provide opportunities for receiving free ITNs from the Ministry of Health particularly at public healthcare facilities in Uganda. Indeed, previous studies have indicated that a significant proportion of pregnant women obtain ITNs from health facilities through ANC visits [11, 24]. Therefore, early and frequent ANC visits should be emphasized during pregnancy in order to provide opportunities for timely delivery of interventions, such as ITNs.

It was revealed that women belonging to the fourth and fifth wealth quintiles were 1.08 times more likely to report using ITNs consistently during pregnancy compared to those in the lowest quintile. This could be because women from a household with a higher wealth quintile are more likely to afford the costs associated with access to maternal health services including transportation costs incurred during ANC visits, and purchasing of ITNs. According to the 2016 Uganda Demographic Health Survey, a significant number of people obtain ITNs from shops/markets [24], implying that women belonging to a lower wealth quintile might not afford to buy these nets [25, 26]. Increase in household wealth as well as economic empowerment of women and their families is expected to increase access to and utilization of malaria prevention interventions among pregnant women including ITNs.

Regarding IPTp, women who had attended at least 4 ANC visits had a 70% higher chance of receiving optimal doses of IPTp-SP compared to those who only attended ANC 3 times or less. Similarly, data from the latest Uganda Demographic and Health survey indicated that the likelihood of taking optimal doses of IPTp-SP was increased among those who attended ANC ≥ 4 times [24]. These findings corroborate those of a study conducted in Ghana where women who attended ANC 4 or more times were found to be positively associated with the uptake of optimal doses of SP [18]. This could be because optimal ANC visits present a good opportunity for timely and appropriate delivery of interventions, including administering IPTp-SP among pregnant women. Therefore, interventions targeted at improving timely ANC attendance are crucial in increasing IPTp uptake hence should be enhanced by the Ministry of Health, Uganda. It was also found that unlike housewives, businesswomen and peasant farmers were more likely to have higher IPTp use, but not insecticide-treated net use. Businesswomen earn from their businesses and can potentially afford buying IPTp or transport to the nearby facilities to get IPTp while peasants may have adequate time to attend the facilities. On the other hand, all households are supplied with nets free of charge through a mass distribution programme targeting pregnant women and children less than 5 years, through a community-based subsidized net distribution program. Its no wonder that no differences existed regardless of the occupation status. Ownership of nets is good indicator that mothers will use them [27].

This study had some limitations for example the outcomes were self-reported hence potential for social desirability bias. Secondly, the study utilized secondary data thus important information on important covariates including the knowledge of the mother on ITN and IPTp was not available. Finally, the study being cross-sectional in nature, hence causal inference cannot be inferred. However, due to the large sample size and wide scope of districts involved in the study, the results can be generalized to the eastern region of Uganda and other similar settings. In addition, the current study findings can inform design of future longitudinal and interventional studies aimed at understanding the causal factors related to optimal IPTp uptake and ITN use in this region.

## Conclusions

The level of uptake of IPTp was below recommended guidelines. Fortunately, the consistent use of ITNs was slightly above the national target of 85%. The study findings highlight the need to encourage optimal ANC visits which may in turn increase the uptake of both preventive measures. Economic empowerment of women and their households is likely to increase uptake of malaria prevention interventions among pregnant women including ITNs.

## Data Availability

The dataset used during the current study is available from the corresponding author on reasonable request.

## Abbreviations

ANC: Antenatal care
HSD: Health sub district
IPTp: Intermittent preventive treatment in pregnancy
IPTp -SP: Intermittent preventive treatment in pregnancy with sulfadoxine pyrimethamine
ITN: Insecticide treated net
PNFP: Private not for profit
PR: Prevalence ratio
WHO: World Health Organization
